# Prevalence and clinical correlates of abnormal lipid metabolism in first-episode and drug-naïve patients with major depressive disorder with abnormal glucose metabolism

**DOI:** 10.1038/s41598-023-35290-6

**Published:** 2023-05-18

**Authors:** Quanfeng Zhu, Guojun Jiang, XiaoE Lang, Zhengchuang Fu, Peng Zhang, Yali Zheng, Xiangyang Zhang

**Affiliations:** 1 Affiliated Xiaoshan Hospital, Hangzhou Normal University, Hangzhou, China; 2 Department of Psychiatry, First Hospital/First Clinical Medical College of Shanxi Medical University, Taiyuan, China; 3 CAS Key Laboratory of Mental Health, Institute of Psychology, Beijing, China; 4 Department of Psychology, University of Chinese Academy of Sciences, 16 Lincui Rd, Beijing, 100101 China

**Keywords:** Depression, Endocrine system and metabolic diseases

## Abstract

Comorbid glucose metabolism abnormalities are very common in patients with major depressive disorder (MDD), and glucose metabolism and lipid metabolism are closely related. However, there are few researches on the incidence and related factors of lipid metabolism abnormalities among MDD patients with comorbid glucose metabolism abnormalities. A cross-sectional study involving 1718 first-episode and drug-naïve (FEDN) MDD patients was conducted. The 17-item Hamilton Depression Scale (HAMD-17), Hamilton Anxiety Rating Scale (HAMA) and Positive and Negative Syndrome Scale (PANSS) positive subscale were utilized to evaluate depressive, anxiety and psychotic symptom, respectively. Serum thyroid function-related parameters, glucose- and lipid-metabolism parameters were measured. The prevalence of abnormal lipid metabolism was significantly higher in FEDN MDD patients with abnormal glucose metabolism than in those without abnormal glucose metabolism (*P* < 0.001). In MDD patients with abnormal glucose metabolism, TSH, FT3 and body mass index (BMI) levels were significantly higher in the abnormal lipid metabolism subgroup than in the non-abnormal lipid metabolism subgroup. Binary logistic regression analysis showed that TSH, FT3 and BMI were the influencing factors of abnormal lipid metabolism in MDD patients with abnormal glucose metabolism (all *P* < 0.05). MDD patients with abnormal glucose metabolism have a high prevalence of abnormal lipid metabolism. Moreover, abnormal glucose metabolism was an independent risk factor for abnormal lipid metabolism in patients with MDD. In addition, thyroid hormone function and BMI may contribute to the co-occurrence of abnormal lipid metabolism in MDD patients with abnormal glucose metabolism.

## Introduction

Among mental diseases, major depressive disorder (MDD) accounts for a very high proportion, which is a severely disabling public health problem worldwide and imposes a very heavy burden on the world^1^. It is characterized by depressed mood and diminished interest. At the same time, it can impair the cognitive function of patients and affect their sleep and appetite^2^. In the United States, a large epidemiological study of MDD in more than 36,000 adults showed high prevalence of 12-month and lifetime MDD which were 10.4% and 20.6%, respectively, and most lifetime MDD was moderate or severe^3^. In China, a study involving more than 28,000 people found that there were more female than male with MDD, more unemployed than employed, and more people living alone than cohabitants^4^. Also, most MDD patients have difficulty with social interaction^4^. Moreover, the treatment rate for MDD patients is very low, with less than 10% of patients receiving adequate treatment^4^. MDD is caused by a combination of different factors, with genetic factors accounting for about 35%^2^. In addition, Environmental factors are also important risk factors for MDD^2^. The pathogenesis of depression is not yet clear, and its neurobiological basis may be related to alterations in monoamines. Besides, some studies have found that the glutamatergic system may also play an important role in the course of depression^5^. Therefore, MDD has been a serious social problem due to its high incidence, relapse rate and low treatment rate^4,6^.

MDD patients have a higher incidence of metabolic syndrome, a higher risk of hyperglycemia and hypertriglyceridemia, which may be associated with the use of antipsychotic medications^7^. Liu and Koponen found that suicide attempts in MDD patients were related to levels of blood glucose, which were significantly higher in patients who had attempted suicide^8,9^. A study by Dong's team found a significant correlation between suicide attempts and blood glucose levels in depressed men, but not in depressed women^10^. The factors influencing suicide risk in depressed patients may be multidimensional and encompass multiple neurobiological as well as psychological underpinnings. For example, previous studies have found that narrative impairment and low psychological resilience may be significant predictors of increased suicidal ideation, while certain religious beliefs may be protective factors for suicidal ideation formation^11,12^. In addition, some other studies have found a higher prevalence of dyslipidemia and abnormal blood glucose in patients with MDD^13–15^. On the one hand, the reason for this is the unhealthy lifestyle habits of the patients; on the other hand, it may be due to the synchronous changes of the metabolic syndrome and depression in certain systems, for instance, hypothalamic–pituitary–adrenal axis, endothelial function and immune system^16^. However, some studies have found the opposite. Jow et al. suggested that patients with MDD had lower serum cholesterol^17^. Messaoud et al’s study had different results, showing that total cholesterol below 3.59 mmol/L may be an indicator of suicide susceptibility in MDD patients^18^. Some studies have revealed that glucose, fatty acid and triglyceride lipoprotein particles intertwine and influence each other at multiple metabolic intersections in multiple tissues, and that glucose and lipid metabolism are inextricably linked through multiple pathways and mechanisms^19^. Wang et al. has shown that hyperglycemia can activate adipogenic genes through multiple pathways, including DNA-dependent protein kinase, preinvasive protein kinase C and AKT-mTOR pathways, they promote adipogenesis by controlling transcription factors and coregulators and lead to elevated lipids^20^. In addition, Barter et al. showed that cholesteryl ester transfer protein inhibitors significantly reduced blood glucose levels in patients whether had diabetes or not, although it's not clear if this is the result of elevated HDL^21^.

As far as we know, this was the first study to explore the lipid metabolism profile of MDD patients with abnormal glucose metabolism. The purpose of our research was to explore the incidence and the related factors of abnormal lipid metabolism in MDD patients with abnormal glucose metabolism.

## Materials and methods

### Participants

All samples were collected from psychiatric outpatients of the First Hospital of Shanxi Medical University from 2015 to 2017. All patients have signed written informed consent, and the study was reviewed and approved by the Ethics Committee of the First Hospital of Shanxi Medical University. The recruitment criteria were as follows :(1) Chinese Han nationality; (2) age range: 18–65; (3) conforming to the diagnostic criteria for MDD in the Diagnostic and Statistical Manual of Mental Disorders, 4th edition (DSM-IV); (4) first-episode and drug-naïve (FEDN); and (5) 17-item Hamilton Depression Scale (HAMD-17) score ≥ 24. Standards of exclusion were :(1) diagnosed with a mental illness other than MDD; (2) infection, immune disease, organic brain disease; (3) history of drug abuse or alcohol dependence; (4) pregnant women; (5) taking any psychotropic drugs.

Adolescence is a special period of growth and development, and the factors influencing suicidal ideation in patients in this period may be more complex; therefore, adolescent patients under the age of 18 were not included in this study. Also, we did not include prenatal depression because the suicidal tendency of these patients may be influenced by the level of hormones in the body during pregnancy and delivery. After excluding several hundred subjects mentioned above, 1718 FEDN MDD patients were enrolled in this study in the aggregate. The study was conducted in accordance with the Declaration of Helsinki and all the items covered were performed in accordance with the relevant guidelines/regulations.

### Clinical interview and assessment

We utilized questionnaires containing race, gender, age, height and weight, marital status, and education background to gather sociodemographic information. We employed the HAMD-17 scale and the Hamilton Anxiety Rating Scale (HAMA) to evaluate severity of depression and anxiety, respectively. And the Positive and Negative Syndrome Scale (PANSS) positive subscale was utilized to evaluate positive psychotic symptoms. Before starting this study, psychiatrists were trained in the use of above scales, and each patient was assessed independently by two psychiatrists.

Patients' anxiety levels were divided into the following levels according to the HAMA total score: (1) no anxiety: HAMA total score ≤ 7; (2) mild anxiety: 8 ≤ HAMA total score ≤ 14; (3) moderate anxiety: 15 ≤ HAMA total score ≤ 23; (4) severe anxiety: HAMA score ≥ 24^22^. Patients with PANSS positive subscale score ≥ 15 were considered to have psychiatric symptoms^10,23^. In addition, the study evaluated whether patients had suicidal behavior in the form of an interview.

### Measurement of physical and biochemical parameters

Fasting biochemical parameters contained fasting blood glucose (FBG), serum total cholesterol (TC), triglyceride (TG), low-density lipoprotein cholesterol (LDL-C), high-density lipoprotein cholesterol (HDL-C), thyroid stimulating hormone (TSH), free triiodothyronine (FT3), free thyroxine (FT4), anti-thyroglobulin antibodies (TgAb) and thyroid peroxidase bntibodies (TPOAb) were measured. FBG > 6.1 mmol/l was considered as abnormal glucose metabolism. Patients with TC ≥ 5.2 mmol/l or TG ≥ 1.7 mmol/l or LDL-C ≥ 3.4 mmol/l or HDL-C < 1.0 mmol/l were considered to have abnormal lipid metabolism. In addition, diastolic and systolic blood pressures were also measured. Patients with blood pressure ≥ 140/90 mmHg were considered hypertension.

### Data analysis

We used the Shapiro–Wilk Test to test all continuous variables to confirm if they fit normal distributions. Continuous variables which fit normal distribution were tested using the Analysis of Variance (ANOVA), rank variables and continuous variables which did not fit normal distribution were tested using Mann–Whitney U Test, and categorical variables were tested using Chi-Square Test. To explore risk factors of abnormal lipid metabolism in MDD patients with abnormal glucose metabolism, we performed univariate analysis of abnormal and non-abnormal lipid metabolism subgroups. We then conducted binary logistic regression analysis on the significantly different factors obtained after univariate analysis, with abnormal lipid metabolism as the dependent variable. Receiver operating characteristic curve (ROC) was utilized to verify the recognition ability of various indexes for abnormal lipid metabolism. Finally, we determined the optimal critical point by calculating the maximum Youden index for each ROC. All statistics were performed on SPSS 25.0. The threshold for significance in this study was 0.05.

### Ethics approval and consent to participate

All patients have signed written informed consent, and the study was reviewed and approved by the Ethics Committee of the First Hospital of Shanxi Medical University.

## Results

### Prevalence of abnormal lipid metabolism of MDD patients with and without abnormal glucose metabolism

The proportion of abnormal glucose metabolism in MDD patients were 13.62% (234/1718). As shown in Table 1, compared with those without abnormal glucose metabolism, MDD patients with abnormal glucose metabolism had higher HAMD score, HAMA score, PANSS positive subscale score, TSH, TGAb, TPOAb, systolic and diastolic blood pressure (all *P* < 0.01). In addition, MDD patients with comorbid abnormal glucose metabolism had a higher proportion of suicidal behavior, psychotic symptom and higher anxiety levels (all *P* < 0.01).

**Table 1:** Sociodemographic and clinical characteristics of MDD with and without abnormal glucose metabolism.

Variable	MDD with abnormal glucose metabolism (N = 234)	MDD without abnormal glucose metabolism (N = 1484)	χ2/z	*P*	Effect sizes Cohen’s d/φ (Phi)
Age	36 (24–47)	34 (23–45)	− 1.1	0.27	0.08
Gender			0.82	0.37	0.02
Male, n (%)	74 (31.6%)	514 (34.6%)			
Female, n (%)	160 (68.4%)	970 (65.4%)			
Education			− 1.93	0.05	0.03
Junior high school, n (%)	69 (29.5%)	344 (23.2%)			
High school, n (%)	100 (42.7%)	660 (44.5%)			
University degree, n (%)	51 (21.8%)	398 (26.8%)			
Master’s degree, n (%)	14 (6.0%)	82 (5.5%)			
Marital status			0.69	0.41	0.02
Single, n (%)	63 (26.9%)	439 (29.6%)			
Married, n (%)	171 (73.1%)	1045 (70.4%)			
Suicide attempters			39.59	< 0.001	0.15
Yes, n (%)	83 (35.5%)	263 (17.7%)			
No, n (%)	151 (64.5%)	1221 (82.3%)			
Anxiety levels			− 5.05	< 0.001	0.05
No anxiety, n (%)	0	0			
Mild anxiety, n (%)	0	6 (0.4%)			
Moderate anxiety, n (%)	163 (69.7%)	1230 (82.9%)			
Severe anxiety, n (%)	71 (30.3%)	248 (16.7%)			
Psychotic symptoms			23.67	< 0.001	0.12
Yes, n (%)	44 (18.8%)	127 (8.6%)			
No, n (%)	190 (81.2%)	1357 (91.4%)			
Abnormal lipid metabolism			22.25	< 0.001	0.11
Yes, n (%)	216 (92.3%)	1177 (79.3%)			
No, n (%)	18 (7.7%)	307 (20.7%)			
Hypertension			17.71	< 0.001	0.1
Yes, n (%)	26 (11.1%)	66 (4.4%)			
No, n (%)	208 (88.9%)	1418 (95.6%)			
HAMD	32 (30–33)	30 (28–32)	− 6.34	< 0.001	0.43
HAMA	22 (20–24)	20 (18–23)	− 5.39	< 0.001	0.39
PANSS positive subscale score	7 (7–11)	7 (7–7)	− 5.81	< 0.001	0.35
BMI, kg/m^2^	24.48 (23.36–26.01)	24.22 (23.21–25.60)	− 1.44	0.15	0.08
Systolic BP, mmHg	124 (118–130)	120 (111–126)	− 6.37	< 0.001	0.48
Diastolic BP, mmHg	78 (74–84)	75 (70–80)	− 6.06	< 0.001	0.44
TC, mmol/L	5.72 (4.97–6.64)	5.12 (4.41–5.86)	− 7.72	< 0.001	0.55
TG, mmol/L	2.30 (1.60–2.98)	1.92 (1.36–2.72)	− 4.62	< 0.001	0.31
HDL-C, mmol/L	1.13 (0.87–1.31)	1.245 (1.04–1.43)	− 5.333	< 0.001	0.36
LDL-C, mmol/L	3.26 (2.70–3.78)	2.90 (2.31–3.49)	− 5.38	< 0.001	0.38
FBG, mmol/L	6.40 (6.23–6.58)	5.20 (4.88–5.60)	− 24.62	< 0.001	2.92
TSH, uIU/mL	7.36 (5.65–9.22)	4.67 (2.94–6.28)	− 12.52	< 0.001	0.96
FT3, pmol/L	4.88 (4.31–5.31)	4.925 (4.39–5.42)	− 1.41	0.16	0.11
FT4, pmol/L	16.63 (14.56–18.88)	16.49 (14.34–18.70)	− 1.06	0.29	0.07
TgAb, IU/L	23.17 (15.38–101.91)	21.27 (14.24–38.51)	− 2.93	0.003	0.12
TPOAb, IU/L	21.39 (12.64–96.28)	17.16 (12.25–32.79)	− 3.19	0.001	0.33

Furthermore, MDD patients with abnormal glucose metabolism had a significantly higher probability of having abnormal lipid metabolism (*P* < 0.001, OR 3.13, 95%CI 1.90–5.15). After controlling for other variables that were significantly different, MDD patients with abnormal glucose metabolism were still significantly more likely to have abnormal lipid metabolism (*P* = 0.04, OR 1.75, 95%CI 1.03–2.99), suggesting that abnormal glucose metabolism was an independent risk factor for abnormal lipid metabolism in MDD patients.

### Differences of abnormal vs. non- abnormal lipid metabolism in MDD patients with abnormal glucose metabolism

Among MDD patients with or without abnormal glucose metabolism, the proportion of abnormal lipid metabolism was 92.31% (216/234) and 79.31% (1177/1484), respectively, which had a significant difference (*P* < 0.001).

Furthermore, as shown in Table 2, among MDD patients with abnormal glucose metabolism, TSH, FT3, and BMI were significantly higher in those with abnormal lipid metabolism than those without abnormal lipid metabolism (all *P* < 0.01).

**Table 2:** Sociodemographic and clinical characteristics of MDD with and without abnormal lipid metabolism.

Variable	MDD with abnormal lipid metabolism (N = 216)	MDD without abnormal lipid metabolism (N = 18)	F/χ2/z	*P*	Effect sizes Cohen’s d/φ (Phi)
Age	35.50 (23.25–47)	39.00 (25.50–46.75)	− 0.61	0.54	0.14
Gender			0.48	0.49	0.05
Male, n (%)	67 (31.0%)	7 (%)			
Female, n (%)	149 (69.0%)	11 (%)			
Education			− 0.2	0.84	0.03
Junior high school, n (%)	64 (29.6%)	5 (27.8%)			
High school, n (%)	91 (42.1%)	9 (50%)			
University degree, n (%)	48 (22.2%)	3 (16.7%)			
Master’s degree, n (%)	13 (6.0%)	1 (5.6%)			
Marital status			0	1	0
Single, n (%)	58 (26.9%)	5 (27.8%)			
Married, n (%)	158 (73.1%)	13 (72.2%)			
Suicide Attempters			0.04	0.84	0.01
Yes, n (%)	77 (35.6%)	6 (33.3%)			
No, n (%)	139 (64.3%)	12 (66.7%)			
Anxiety levels			− 0.29	0.77	0.04
No anxiety, n (%)	0	0			
Mild anxiety, n (%)	0	0			
Moderate anxiety, n (%)	151 (69.9%)	12 (66.7%)			
Severe anxiety, n (%)	65 (30.1%)	6 (33.3%)			
Psychotic symptoms			0.01	0.94	0.01
Yes, n (%)	40 (18.5%)	4 (22.2%)			
No, n (%)	176 (81.5%)	14 (77.8%)			
Hypertension			0	1	0
Yes, n (%)	24 (11.1%)	2 (11.1%)			
No, n (%)	192 (88.9%)	16 (88.9%)			
HAMD	32 (30–33)	31 (26.75–33.25)	− 1.12	0.26	0.27
HAMA	22 (20–24)	20.50 (18–25.50)	− 0.47	0.64	0.08
PANSS positive subscale score	7 (7–11)	7 (7–10.75)	− 0.39	0.7	0.04
BMI, kg/m^2^	24.59 (23.49–26.04)	23.425 (22.33–24.26)	− 2.88	0.004	0.66
Systolic BP, mmHg	123.94 ± 9.86	122.61 ± 10.58	0.35	0.59	0.13
Diastolic BP, mmHg	78 (74–84)	77 (71.50–80.50)	− 1.44	0.15	0.35
TC, mmol/L	5.92 (5.31–6.70)	4.62 (3.86–4.78)	− 5.44	< 0.001	1.73
TG, mmol/L	2.38 (1.81–3.17)	1.24 (1.06–1.55)	− 5.83	< 0.001	1.94
HDL-C, mmol/L	1.09 (0.86–1.30)	1.26 (1.17–1.58)	− 3.39	0.001	0.94
LDL-C, mmol/L	3.33 ± 0.95	2.56 ± 0.37	8.27	< 0.001	1.18
FBG, mmol/L	6.4 (6.23–6.58)	6.47 (6.19–6.89)	− 0.38	0.71	0.23
TSH, uIU/mL	7.39 (5.83–9.25)	4.66 (2.17–8.23)	− 2.72	0.007	0.76
FT3, pmol/L	4.92 (4.40–5.32)	4.27 (4.09–4.73)	− 2.73	0.006	0.65
FT4, pmol/L	16.63 (14.61–18.88)	17.01 (14.17–18.99)	− 0.08	0.94	0.01
TgAb, IU/L	23.19 (15.61–120.36)	19.84 (13.46–40.96)	− 1.3	0.19	0.38
TPOAb, IU/L	22.53 (12.66–97.42)	14.47 (11.93–62.30)	− 1.21	0.23	0.08

### Risk factors for abnormal lipid metabolism in MDD patients with abnormal glucose metabolism

The results of the binary logistic regression analysis after including the variables with significant differences in univariate analysis showed that, the risk factors for abnormal lipid metabolism included TSH (B = 0.29, *P* = 0.02, OR 1.34, 95%CI 1.12–1.61), FT3 (B = 0.91, *P* = 0.02, OR 2.49, 95%CI 1.17–5.27) and BMI (B = 0.28, *P* = 0.02, OR 1.32, 95%CI 1.05–1.66).

**Figure 1:**
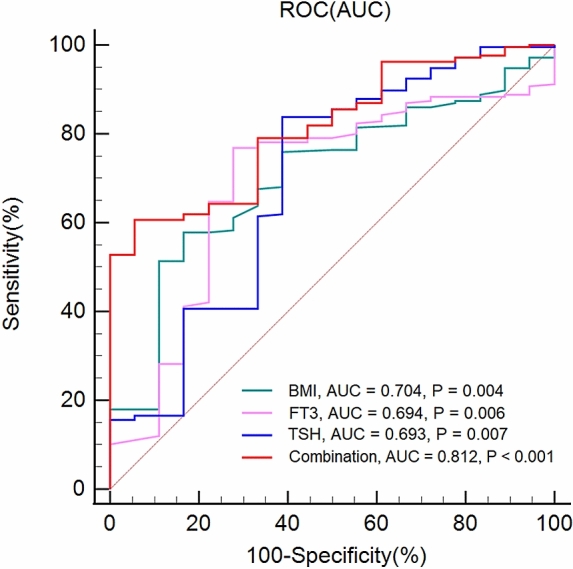
The ability of related factors to distinguish abnormal lipid metabolism in MDD patients with abnormal glucose metabolism.

The area under the ROC curve (AUC) of TSH, FT3 and BMI were 0.693, 0.694 and 0.704, respectively. Furthermore, when we combined the three risk factors, we obtained a higher AUC of 0.812 (Fig. 1).

### Determination of the optimal critical value of risk factors

The maximum Youden indexes of TSH, FT3 and BMI were 0.45, 0.49 and 0.41, respectively. And the optimal critical values of them were 4.79, 4.38 and 24.29, respectively. Taken together, if TSH ≥ 4.79 μIU/ml, FT3 ≥ 4.38 pmol/L and BMI ≥ 24.29 kg/m^2^, the probability of abnormal lipid metabolism in MDD patients with abnormal glucose metabolism was more than 81.2%.

## Discussion

As far as we know, our study is the first to explore the prevalence and risk factors for dyslipidemia in a large sample size of FEDN MDD patients. Our major findings are: (1) the incidence of abnormal lipid metabolism was remarkably higher in patients with FEDN MDD with abnormal glucose metabolism than in patients without abnormal glucose metabolism; (2) among FEDN MDD patients with abnormal glucose metabolism, risk factors for abnormal lipid metabolism included TSH, FT3, and BMI; and (3) if the three conditions of TSH ≥ 4.79 μIU/ml, FT3 ≥ 4.38 pmol/L and BMI ≥ 24.29 kg/m^2^ were met in FEDN MDD patients with abnormal glucose metabolism, their prevalence of abnormal lipid metabolism in patients exceeded 81.2%.

To date, there were few researches on the prevalence of abnormal lipid metabolism and its risk factors in MDD patients combined with abnormal glucose metabolism. Some studies have reported a higher incidence of metabolic syndrome in MDD patients than healthy population. Vancampfort’s study reported that MDD patients were more prone to metabolic syndrome than healthy controls of the same age and gender, which were significantly associated with antipsychotic use, regardless of sex, age, geographic area, smoking, and other factors^7^. Kahl et al. also suggested that metabolic syndrome was more than twice as prevalent in MDD patients than in healthy population of the same age (41.0% vs. 17.0%), in particular, the levels of FBG and TG were remarkably higher in MDD patients, and the increase correlated with the severity of depression^24^. However, the opposite results were obtained by Jow's team, who found that cholesterol levels were lower in MDD patients and that the degree of depression in MDD patients were inversely correlated with cholesterol levels^17^. On the one hand, these differences may be due to heterogeneity between samples, such as patients at different stages of the disease, or slightly different diagnostic criteria for HAMD scores in MDD in different studies. These factors may lead to different results. On the other hand, it may be due to the different antipsychotic or antidepressant medications that patients were taking, which may have a significant effect on lipids and blood glucose.

In the present study, patients with FEDN MDD were recruited to reduce interference of different disease progression or medications on our study. In addition, we explored for the first time the prevalence and risk factors of abnormal lipid metabolism in FEDN MDD patients with abnormal glucose metabolism. We found that the prevalence of abnormal lipid metabolism was remarkably higher in MDD patients with abnormal glucose metabolism than those without abnormal glucose metabolism. Among patients with MDD, abnormal glucose metabolism was an independent risk factor for abnormal lipid metabolism.

A variety of factors, such as genetic factors, gut microbiota and thyroid function, can affect lipid metabolism in humans. The influence of genetic factors on lipid metabolism is more common in mutations in lipid regulators and proteins and other metabolic pathways^25^. The effect of intestinal flora on lipids may be mediated by metabolites produced by the flora and factors derived from pro-inflammatory bacteria^26^. The effects of thyroid hormones on lipid levels in humans occur throughout the entire process of lipid metabolism, including lipid production, disposal, and excretion^27,28^. Several studies have shown that thyroid hormones may mediate lipid synthesis by autophagy during transcription and other processes^29^.

However, factors influencing lipid metabolism in MDD patients, even those with abnormal glucose metabolism, have not been reported. Our study showed that TSH, FT3 and BMI were risk factors for abnormal lipid metabolism in patients with MDD. It is similar to the results of Wang’s team, who reported that elevated TSH levels were accompanied by elevated TC and LDL-C levels^30^. Furthermore, our results were validated by the study of Delitala et al., who reported that lipid metabolic status worsened with increasing TSH levels^31^. Also, Lei et al. found that levels of TC, TG, and LDL-C were significantly lower in hypothyroid patients^32^. The association between BMI and abnormal lipid metabolism is easily explained. The higher the BMI, the more severe the obesity, which itself is to some extent an indication of elevated lipid levels. At another level, obesity leads to the accumulation of triglycerides in the liver, leading to dysregulation of hepatic metabolism, which affects lipid metabolism^33^.

After calculating the maximum Youden index and optimal cut-off value of each risk factor, we learned that when levels of TSH, FT3 and BMI of FEDN MDD patients with abnormal glucose metabolism reached 4.79 μIU/ml, 4.38 pmol/L, 24.29 kg/m2 or higher, their prevalence of abnormal lipid metabolism was more than 81%. Due to the adverse effects of antipsychotics and antidepressants on glucose and lipid metabolism, we would expect this proportion to be higher in MDD patients taking medication.

Although most clinical practitioners are aware of the relatively large correlation between glucose metabolism and lipid metabolism, it remains less well established whether this correlation is clear in depressed patients. Our study confirms the significant correlation between abnormal glucose metabolism and abnormal lipid metabolism in FEDN MDD patients, and the lipid levels in patients taking glucose-lowering medications or with abnormal glucose metabolism deserve even more attention in terms of clinical and nursing care. And because of the common adverse effects of antipsychotics and antidepressants on patients' metabolism, the glycolipid metabolic capacity of patients on long-term medications should be of greater concern to clinicians and nurses. In addition, an important concern is the correlation between abnormal lipid metabolism and thyroid hormones. Although high thyroid hormone levels are not necessarily clinically significant, it is important for clinicians and nurses to recognize this potential association to facilitate timely and comprehensive evaluation of patients.

Our research has some limitations. First, we could not explore the causal relationship between abnormal lipid metabolism and other factors. Secondly, the study participants we recruited were all Han Chinese populations from the same region, therefore, our research results may differ somewhat from other regions. Thirdly, this study did not perform additional staging of MDD and did not assess the cognitive function and intellectual status of patients, which may have lost some meaningful results.

## Conclusions

In conclusion, our study revealed that FEDN MDD patients with abnormal glucose metabolism had a higher prevalence of abnormal lipid metabolism, and the associated risk factors of abnormal lipid metabolism were levels of TSH, FT3, and BMI. In the future, we hope to conduct longitudinal studies to explore the causal relationship between these factors.

## Data Availability

The data that support the findings of this study are available from the corresponding author upon reasonable request.

## Abbreviations

MDD: Major depressive disorder
DSM-IV: Diagnostic and statistical manual of mental disorders, 4th edition
FEDN: First-episode and drug-naïve
HAMD-17: 17-Item Hamilton depression scale
HAMA: Hamilton anxiety rating scale
PANSS: Positive and negative syndrome scale
FBG: Fasting blood glucose
TC: Total cholesterol
TG: Triglyceride
LDL-C: Low-density lipoprotein cholesterol
HDL-C: High-density lipoprotein cholesterol
TSH: Thyroid stimulating hormone
FT3: Free triiodothyronine
FT4: Free thyroxine
TgAb: Anti-thyroglobulin antibodies
TPOAb: Thyroid peroxidase bntibodies
ANOVA: Analysis of variance
ROC: Receiver operating characteristic
AUC: Area under the receiver operating characteristic curve
